# Community Awareness, Knowledge, Attitudes, and Perceptions Toward Viral Disease Outbreaks in Dar es Salaam, Tanzania

**DOI:** 10.1002/puh2.70232

**Published:** 2026-04-13

**Authors:** Mohammed Elmogiera Fadlallh Elsayed, Emmanuel J. Magandi, Sima Rugarabamu

**Affiliations:** ^1^ University of Medical Sciences & Technology (UMST) Khartoum Sudan; ^2^ Department of Internal Medicine Muhimbili National Hospital—Mloganzila Dar es Salaam Tanzania; ^3^ Clinical Research Training & Consultancy Unit Muhimbili National Hospital Dar es Salaam Tanzania; ^4^ Department of Microbiology and Immunology Muhimbili University of Health and Allied Sciences Dar es Salaam Tanzania

**Keywords:** attitudes, knowledge, misinformation, practices, public health preparedness, stigma, Tanzania, vaccine hesitancy, viral outbreaks

## Abstract

**Background:**

The frequency and scale of viral disease outbreaks are increasing globally, with communities in sub‐Saharan Africa disproportionately affected due to structural, social, and health‐system vulnerabilities. Community knowledge, attitudes, and perceptions (KAP) play a critical role in outbreak prevention, control, and response. This study assessed KAP related to viral disease outbreaks among adults in Dar es Salaam, Tanzania, following recent outbreaks, including COVID‐19, Mpox, and Marburg virus disease.

**Methods:**

A cross‐sectional study was conducted among 104 adult visitors attending Muhimbili National Hospital between October and November 2025. Participants were recruited using consecutive sampling. Data were collected using a structured questionnaire capturing sociodemographic characteristics, knowledge of viral disease transmission and treatment, intended preventive practices, sources of health information, stigma‐related attitudes, and vaccine perceptions. Data were analyzed using descriptive statistics, chi‐square (*χ* ^2^) tests, and regression analyses to explore associations between KAP domains and participant characteristics.

**Results:**

General awareness of viral disease outbreaks was high (87.5%); however, substantial gaps were identified in specific knowledge. Only 33.7% of participants correctly recognized that antibiotics are ineffective against viral infections, and fewer than half (46.2%) rejected the misconception that viral diseases are spread only by foreigners. A notable belief‐action gap was observed regarding vaccination: Although 71.2% believed vaccines are effective, only 59.6% expressed willingness to be vaccinated. Stigmatizing attitudes were prevalent, with 81.7% of respondents reporting discomfort with welcoming a recovered outbreak survivor into their home. Social media platforms were the primary source of outbreak‐related information (51.9%), whereas official national public health communication channels were among the least utilized (22.1%). Knowledge levels showed no significant association with sociodemographic characteristics; however, lower educational attainment was significantly associated with higher levels of stigma (*p* = 0.02). Vaccine hesitancy was primarily driven by concerns about vaccine efficacy and fear of side effects rather than overall knowledge deficits.

**Conclusion:**

Despite high general awareness of viral disease outbreaks, this study identifies critical weaknesses in specific knowledge, persistent stigma, and vaccine hesitancy that pose challenges for urban healthcare‐seeking populations in Dar es Salaam. These findings underscore the need for a strategic shift from broad awareness campaigns to precision public health interventions, including targeted myth‐busting, community‐led anti‐stigma initiatives, and proactive digital engagement to address misinformation, build trust, and strengthen collective outbreak response.

## Graphical abstract

High awareness of viral outbreaks in Dar es Salaam coexists with critical knowledge gaps, stigma, and vaccine hesitancy driven by trust and safety concerns. Targeted myth correction, anti‐stigma initiatives, and strategic digital health communication are essential to strengthen community preparedness and outbreak response.

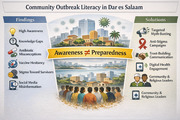

## 1 Introduction

The global health landscape is increasingly marked by the emergence and re‐emergence of viral disease outbreaks [1]. The incidence of such outbreaks has risen dramatically over recent decades, a trend exacerbated by urbanization, climate change, and human encroachment into wildlife habitats [2]. These health emergencies place a catastrophic strain on health systems and economies, with low‐income and middle‐income countries (LMICs) in sub‐Saharan Africa bearing a disproportionate burden [3].

Tanzania, with its specific ecological and demographic profile, is a recognized hotspot for zoonotic diseases and infectious disease transmission [4]. The country has experienced outbreaks of cholera, dengue, Rift Valley fever, and, more recently, Marburg virus disease and mpox, underscoring its persistent vulnerability to viral threats [5, 6]. The critical role of an informed and engaged community in outbreak control is well‐established; the success of measures from case detection and isolation to vaccine uptake hinges on public knowledge, trust, and cooperation [7, 8].

Although previous research in Tanzania, such as a study on viral hemorrhagic fevers (VHFs), revealed alarmingly low community knowledge and high‐risk practices in specific disease contexts [9], a comprehensive assessment of general “outbreak literacy” in a major urban African population in the post‐COVID‐19 era is lacking. The pandemic led to an unprecedented, and often conflicting, level of public exposure to health information, potentially cementing myths and eroding trust. This study therefore sought to assess the current state of knowledge, attitudes, and perceptions (KAP) regarding viral disease outbreaks among adults in Dar es Salaam, Tanzania's largest city.

We hypothesized that although general awareness would be high, critical knowledge gaps regarding transmission and treatment, as well as significant behavioral barriers (vaccine hesitancy and stigma), would persist and would be linked more to trust and educational factors than to factual knowledge alone. This study was guided by the Health Belief Model (HBM) and principles of risk communication theory, which propose that health behaviors are influenced by perceived susceptibility, perceived severity, perceived benefits and barriers, cues to action, and trust in information sources. These frameworks informed the study objectives, questionnaire design, and interpretation of attitudes toward vaccination, stigma, and preventive behaviors.

## 2 Methods

### 2.1 Study Design and Setting

A descriptive cross‐sectional study was conducted between October and November 2025 at Muhimbili National Hospital (MNH) in Dar es Salaam, Tanzania. As the nation's primary tertiary referral hospital, MNH serves a diverse patient population from across the city and country. Its visitor population, representing a cross‐section of individuals from all five districts of Dar es Salaam attending to patients, was selected as a practical and diverse proxy for a community‐based sample of the city's adult residents, avoiding the potential biases of sampling only patients. Although visitors to a tertiary hospital may have slightly higher health awareness than the general population, this sampling frame provides a practical and accessible proxy for urban residents, capturing individuals from all socioeconomic districts of Dar es Salaam during routine hospital visitation.

Although MNH visitors represent individuals from all districts of Dar es Salaam, the sample was not intended to be statistically representative of the entire city population but rather to provide exploratory insights into outbreak literacy among urban healthcare‐seeking adults.

### 2.2 Participants

The study population consisted of adult visitors (aged ≥18 years) at MNH. Individuals who identified as healthcare professionals or public health officials were excluded to capture layperson knowledge. A consecutive sampling technique was employed. Over predetermined shifts, research assistants systematically approached every third eligible adult in high‐traffic areas (e.g., main waiting bays and hospital entrances). Of 106 individuals approached, 104 provided complete responses and were included in the final analysis.

As an exploratory cross‐sectional study conducted over a defined 2‐month period, the sample size was determined by feasibility and participant flow rather than formal power calculation. The final sample size is consistent with similar hospital‐based KAP studies [10] and was sufficient to identify key knowledge gaps and attitudinal patterns.

### 2.3 Data Collection Tool and Procedure

Data collection was conducted through face‐to‐face interviews using a structured questionnaire that was pre‐tested for clarity and reliability. The instrument, available in both Kiswahili and English, was developed following a review of pertinent literature [9, 10] and subsequently adapted to ensure cultural and contextual relevance. It was organized into six thematic sections: (A) sociodemographic characteristics; (B) general knowledge of viral diseases, including awareness, symptoms, and common transmission myths; (C) knowledge of specific viruses and their transmission routes; (D) intended preventive practices; (E) information sources and risk perception, the latter measured using 5‐point Likert scales; and (F) attitudes towards vaccines and disease survivors. Observed practices were not measured because the study was conducted outside an active outbreak context; therefore, intended practices were used as proxies for behavioral readiness.

The questionnaire was translated into Kiswahili by a certified translator and back‐translated to ensure conceptual equivalence. It was pretested on 10 individuals at a different health facility. Cronbach's alpha for the attitude scales was calculated to be above 0.7, indicating good internal consistency. Written informed consent was obtained from all participants.

### 2.4 Data Analysis

Data were cleaned, coded, and entered into Microsoft Excel before being imported into SPSS V.26 for analysis. Descriptive statistics (frequencies, percentages, means, and SD) were used to summarize the data. A composite knowledge score was calculated from 11 key binary questions (1 point for correct, 0 for incorrect/don't know). Participants were then dichotomized into “Good knowledge” (score ≥8) and “Lower knowledge” (score <8) categories for analysis. The ≥8/11 threshold represents approximately 70% correct responses and was selected to provide a meaningful distinction between higher and lower knowledge levels, consistent with thresholds used in similar knowledge assessment studies [10].

*χ* ^2^ tests and ANOVA were used to examine associations between sociodemographic variables and knowledge or attitudinal outcomes. Preliminary multivariable logistic and linear regression models were built to identify independent predictors of vaccine hesitancy and stigma, controlling for key demographics. Variables included in multivariable models were selected on the basis of theoretical relevance and bivariate associations (*p* < 0.20). Model assumptions were evaluated using the Hosmer–Lemeshow goodness‐of‐fit test and residual analysis. Multicollinearity was assessed using variance inflation factors (VIF), with values below 5 considered acceptable. A *p* value of <0.05 was considered statistically significant. For multivariable regression models, categorical variables were coded with the following reference categories: for sex, male; for education, primary or less; and for occupation, shopkeeper/businessperson.

### 2.5 Patient and Public Involvement

Patients or the public were not involved in the design, or conduct, or reporting, or dissemination plans of our research.

Ethical approval was obtained from the Muhimbili National Hospital Institutional Review Board (MNH/IRB/Vol. IX/2025/031).

## 3 Results

### 3.1 Sociodemographic Characteristics of the Respondents

A total of 104 participants were enrolled in the study. The mean age was 36.0 years (SD ±13.0). The sample was predominantly male (56.7%). The largest proportion of respondents (52.9%) were in the 18–35 age group. In terms of education, most participants had attained a secondary education (40.4%), and the most common occupation was shopkeeper or businessperson (38.5%). Participants were recruited from all five districts of Dar es Salaam. The sociodemographic characteristics of the participants are detailed in Table 1. Chi‐square tests revealed no statistically significant associations between any of these characteristics and the level of knowledge about viral disease outbreaks (all *p* values >0.05).

**TABLE 1:** Sociodemographic characteristics of participants (*n* = 104).

Variable	Category	Frequency (*n*)	Percentage	*p* value
**Sex**	Male	59	56.7	
	Female	45	43.3	0.68
**Age group (years)**	18–35	55	52.9	
	36–55	34	32.7	0.37
	≥56	15	14.4	
**Education level**	Primary or less	29	27.9	
	Secondary	42	40.4	0.31
	College/University	33	31.7	
**Occupation**	Shopkeeper/Business	40	38.5	
	Unemployed	22	21.2	
	Skilled/Manual labor^a^	21	20.2	0.85
	Student	7	6.7	
	Other^b^	14	13.5	

^a^ Skilled/Manual labor: Includes teacher, public administration, construction worker, and transport service provider.

^b^ Other: Includes all remaining occupations not specified in the above categories.

### 3.2 Knowledge Regarding Viral Disease Outbreaks

Awareness of viral outbreaks was high, with 87.5% of respondents having heard of one before. Knowledge of symptoms was also strong; 76.0% knew viruses could cause bleeding and 86.5% recognized rashes as a symptom. However, profound misconceptions were identified. Only 46.2% correctly rejected the myth that “only foreigners/people from elsewhere can spread the virus,” and a mere 37.5% knew that a healthy, asymptomatic person could transmit infection. Critically, only 33.7% of participants knew that antibiotics are ineffective against viral diseases. Although 80.8% understood the value of avoiding crowds, only 31.7% reported they would not delay seeking care or isolating if they developed symptoms. Most participants (92.3%; 96/104) understood the protective value of handwashing and mask use during outbreaks (Table 2).

**TABLE 2:** Participant knowledge of viral disease outbreaks (*N* = 104).

Knowledge item	% Correct response (*n*)
Heard of a viral disease outbreak before	87.5 (91)
Aware of viral outbreaks in Tanzania beyond COVID‐19	68.3 (71)
Viruses can cause internal/external bleeding	76.0 (79)
Viruses can cause rashes	86.5 (90)
*Myth:* Only foreigners can spread the virus (correct: no)	46.2 (48)
*Myth:* A healthy person cannot infect another (correct: no)	37.5 (39)
Avoiding crowded places prevents spread	80.8 (84)
*Myth:* Traditional medicines are effective (correct: no)	52.9 (55)
*Myth:* Antibiotics can treat viral diseases (correct: no)	33.7 (35)
Would not delay care until symptoms are severe	31.7 (33)
Handwashing and masks are effective during outbreak	92.3 (96)

### 3.3 Preventive Practices and Information Sources

The most frequently cited intended actions to limit spread were using masks/gloves (52.9%), avoiding crowded places (48.1%), and handwashing (48.1%). A notable finding was that 9.6% of all respondents stated they would take no action, relying instead on the belief that “God is the protector” (Table 3).

**TABLE 3:** Intended preventive actions during an outbreak (multiple responses allowed, *N* = 104).

Intended action	% Selecting (*n*)
Use mask and gloves	52.9 (55)
Avoid crowded places	48.1 (50)
Wash hands frequently	48.1 (50)
Avoid contact with others	45.2 (47)
Avoid travel to outbreak areas	38.5 (40)
Avoid mosquito bites	25.0 (26)
Take no action (God is the protector)	9.6 (10)

**FIGURE 1:**
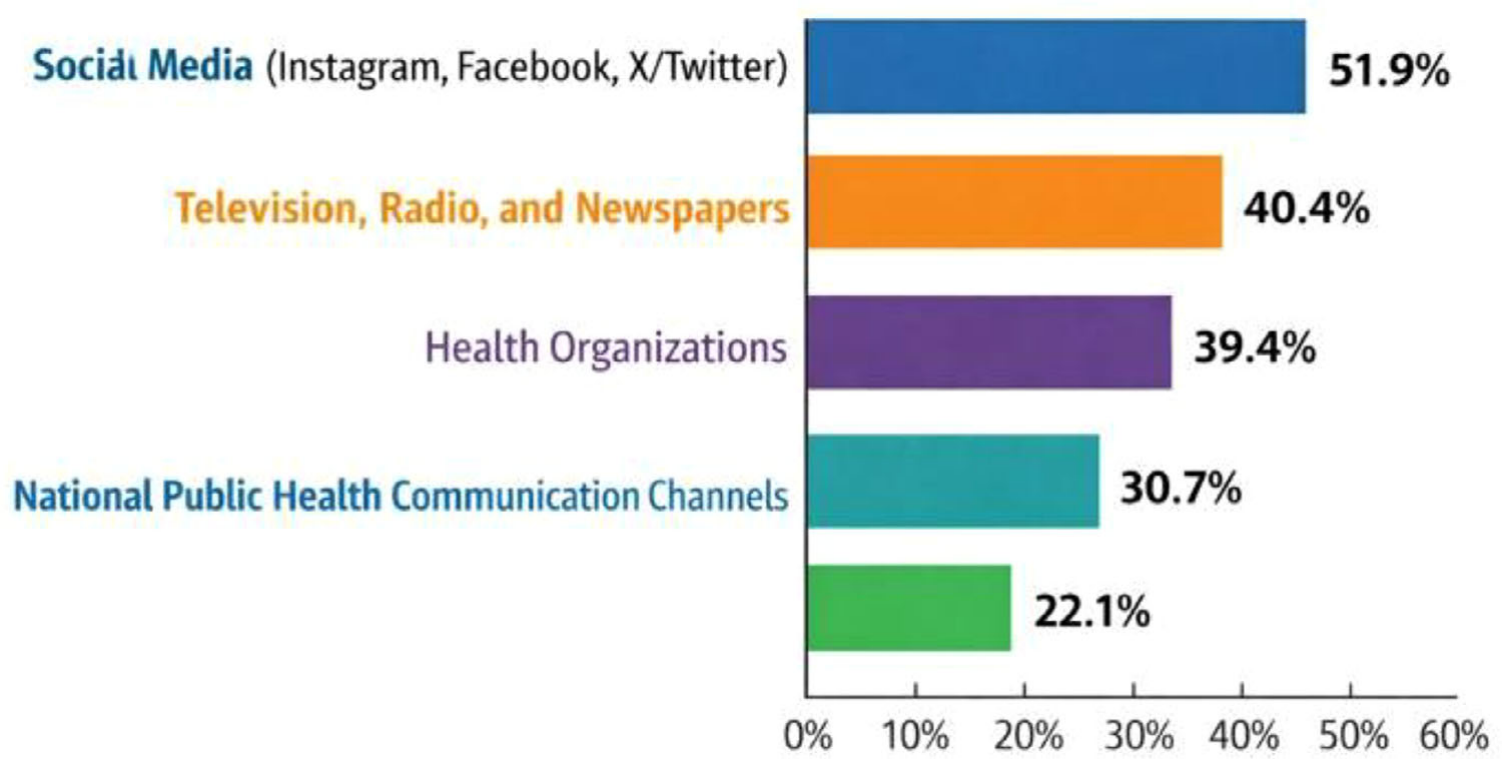
Primary sources of information on viral disease outbreaks among study participants in Dar es Salaam, Tanzania (*N* = 104). Respondents could select multiple sources.

When asked about their primary sources of information on viral disease outbreaks, respondents most frequently cited social media platforms such as Instagram, Facebook, and X/Twitter (51.9%), followed by television, radio, and newspapers (40.4%) and health organizations (39.4%). In contrast, national public health communication channels were reported less frequently (22.1%) (Figure 1).

### 3.4 Attitudes, Stigma, and Vaccine Perceptions

Survey respondents indicated a strong desire for more information about viral outbreaks (mean score 4.19 ± 1.01 on a 5‐point scale). However, the mean level of comfort with welcoming a disease survivor into one's home was low (2.42 ± 1.27), indicating significant stigma within the community. Although belief in vaccine efficacy was relatively high (3.98 ± 1.07), the stated willingness to take a vaccine during an outbreak was lower (3.67 ± 1.26), revealing a notable belief‐action gap (Table 4).

**TABLE 4:** Risk perception, stigma, and vaccine attitudes among study participants (*N* = 104).

Statement	Mean	SD	%Agree/Strongly agree*
I would like to be more informed about viral outbreaks	4.19	1.01	82.7
I believe I am at risk of contracting a viral disease	2.91	1.42	36.5
I would be comfortable welcoming a survivor into my home	2.42	1.27	18.3
Vaccines are effective in preventing viral diseases	3.98	1.07	71.2
I would be willing to take a vaccine if available	3.67	1.26	59.6

### 3.5 Factors Associated With Key Outcomes

#### 3.5.1 Factors Associated With Comprehensive Knowledge

No sociodemographic characteristic, including age, sex, education level, or occupation, showed a statistically significant association with the composite “Good Knowledge” score (all *p* > 0.05). A non‐significant trend suggested that participants who cited the World Health Organization (WHO) as an information source had a higher proportion of good knowledge scores (45.2%) compared to those who did not (32.8%) (*χ* ^2^ = 3.08, *p* = 0.08).

#### 3.5.2 Factors Associated With Vaccine Hesitancy

The disparity between the high belief in vaccine efficacy and the lower willingness for vaccination was strongly linked to specific concerns. Participants who reported “lack of belief in efficacy” were significantly more likely to be hesitant (*χ* ^2^ = 22.1, *p* < 0.001), and those who cited “fear of side effects” also showed significantly reduced willingness to be vaccinated (*χ* ^2^ = 11.2, *p* = 0.003). A preliminary multivariable logistic regression model adjusting for age and sex confirmed both as strong, independent predictors of hesitancy: lack of belief in efficacy (AOR: 0.15, 95% CI: 0.06–0.38) and fear of side effects (AOR: 0.28, 95% CI: 0.12–0.65). In contrast, the general knowledge score was not a significant predictor (*p* = 0.42).

#### 3.5.3 Factors Associated With Stigma

Stigma (measured as discomfort welcoming a survivor) was significantly correlated with socioeconomic and belief‐based factors. Participants with lower levels of formal education demonstrated higher levels of stigma (ANOVA *F* = 4.12, *p* = 0.02), showing a clear inverse educational gradient. Belief in the efficacy of traditional medicines against viral diseases was also associated with higher stigma in bivariate analysis (*t* = −2.10, *p* = 0.04). However, when traditional medicine belief was included in a preliminary linear regression model together with education, the association was attenuated (*p* = 0.15), suggesting that lower educational attainment may be the more fundamental correlate of stigmatizing attitudes.

## 4 Discussion

This study assessed community awareness, knowledge, attitudes, and perceptions regarding viral disease outbreaks in Dar es Salaam, Tanzania. Although general awareness of viral outbreaks was high, the findings reveal substantial gaps in specific knowledge, alongside behavioral and attitudinal challenges that may undermine effective outbreak preparedness and response.

Overall knowledge levels were lower than those reported in previous studies on epidemic‐prone diseases conducted in comparable settings [9, 10]. Notably, only about one‐third of respondents correctly identified that antibiotics are ineffective against viral infections, and fewer than half rejected the misconception that viruses are spread only by foreigners or people from outside the community. In addition, less than 40% recognized that asymptomatic individuals can transmit viral infections. These misconceptions indicate that, despite heightened public attention following recent pandemics, fundamental concepts regarding viral transmission and treatment remain poorly understood in urban populations.

Patterns of information‐seeking behavior further illuminate important gaps in public health communication. Social media platforms were the most frequently reported sources of outbreak‐related information, followed by television, radio, and newspapers, whereas official national public health communication channels were cited less often. This finding is consistent with evidence from other African contexts where digital and mass media dominate health information dissemination [10, 11]. Importantly, many official public health messages are themselves disseminated through social media platforms, which may make it difficult for individuals to distinguish institutional sources from informal or unverified content. These findings underscore the need for public health authorities to strategically leverage widely used media platforms while strengthening message clarity, credibility, and visibility, a challenge further highlighted by recent reviews of misinformation dynamics during African outbreaks [12].

Vaccine attitudes revealed a notable discrepancy between belief and intended behavior. Although a majority of participants expressed confidence in vaccine effectiveness, a substantially smaller proportion reported willingness to be vaccinated during an outbreak. Statistical analyses demonstrated that vaccine hesitancy was driven primarily by concerns about efficacy and fear of side effects rather than by inadequate general knowledge. This pattern aligns with findings from other settings, where trust, perceived safety, and confidence in institutions play a more decisive role in vaccine acceptance than factual knowledge alone [13, 14, 15]. From a theoretical perspective, the disconnect between knowledge and preventive behaviors aligns with the HBM, suggesting that perceived risks, trust in institutions, and perceived barriers influence preventive actions more strongly than factual knowledge alone [11, 12, 13]. Social trust, stigma, and cultural beliefs may mediate outbreak response behaviors, underscoring the importance of culturally grounded risk communication strategies [13].

Stigmatizing attitudes toward disease survivors were also prominent. Most respondents reported discomfort with welcoming a survivor into their home, reflecting persistent fear and social exclusion associated with infectious diseases. Such stigma can discourage timely care‐seeking, hinder contact tracing, and weaken community solidarity during outbreaks. The observed association between lower educational attainment and higher levels of stigma suggests that social vulnerability may amplify stigmatizing beliefs, even when general awareness is relatively high [15].

A small but meaningful proportion of respondents reported that they would take no preventive action during an outbreak, citing reliance on divine protection. This finding highlights the complex interplay between scientific understanding, cultural beliefs, and religious perspectives in shaping health behaviors. Similar dynamics have been reported in other settings, where faith‐based interpretations influence responses to public health threats [15, 16]. These insights point to the importance of engaging religious and community leaders as partners in outbreak preparedness and response efforts.

Taken together, the findings indicate that high general awareness alone does not translate into effective outbreak preparedness. Persistent misconceptions, vaccine hesitancy driven by trust concerns, reliance on informal information sources, and stigma toward survivors represent critical barriers that must be addressed through more nuanced and community‐centered communication strategies [17, 18]. Although the study sample was hospital‐based and not population‐representative, the findings provide important exploratory insights into outbreak literacy gaps among urban healthcare‐seeking populations.

These findings carry several implications for policy and practice within Tanzania's health system. To address the predominant reliance on informal digital information sources, official public health messaging could benefit from strategic collaboration with popular social media influencers and community leaders, thereby enhancing message credibility and reach. In response to the identified drivers of vaccine hesitancy, namely concerns about efficacy and fear of side effects, engagement strategies that prioritize transparency and dialogue may prove more effective than conventional information campaigns. Community question and answer sessions facilitated by trusted local health workers represent one such approach. Addressing stigma, a persistent barrier to outbreak response, calls for community‐led initiatives that integrate recovered individuals into educational efforts to humanize the experience of disease and reduce social exclusion. Embedding these approaches within Tanzania's existing outbreak response frameworks, informed by lessons drawn from the recent Marburg virus disease response, could contribute to strengthening public trust and fostering greater community resilience.

### 4.1 Limitations

This study has several limitations. The hospital‐based sampling and modest sample size limit generalizability beyond urban healthcare‐seeking populations. Selection bias may have occurred, as visitors to a tertiary hospital may differ from the general community in health awareness. Social desirability bias may have influenced responses related to preventive behaviors and stigma. Additionally, translation and interviewer‐administered surveys may introduce subtle interpretation differences despite rigorous translation procedures. Nonetheless, the findings provide important exploratory insights into outbreak literacy and behavioral barriers in an urban Tanzanian context.

## 5 Conclusion

This study demonstrates a persistent gap in Dar es Salaam between high general awareness of viral disease outbreaks and deficiencies in specific knowledge, accompanied by behavioral challenges such as vaccine hesitancy and stigma. Misconceptions about viral transmission and treatment remain common, and willingness to accept vaccination is influenced more by trust and safety concerns than by knowledge levels.

The predominance of social media and mass media as sources of outbreak‐related information, coupled with lower reliance on official national communication channels, highlights an opportunity to enhance the reach, credibility, and effectiveness of public health messaging through platforms that communities already use and trust. Educational attainment was associated with stigma but not with general knowledge, suggesting that misinformation cuts across social groups and requires broad‐based, inclusive interventions.

These findings highlight the need to move beyond generalized awareness campaigns toward more targeted, participatory, and platform‐aware public health communication strategies. Such approaches should prioritize correction of specific misconceptions, transparent engagement around vaccine safety, stigma reduction through community dialogue, and proactive dissemination of accurate information across both digital and traditional media. Strengthening these areas will be essential for building informed, cohesive, and resilient urban communities in Dar es Salaam and similar settings facing recurrent viral disease threats.

## Author Contributions

**Mohammed Elmogiera Fadlallh Elsayed**: conceptualization, methodology, investigation, formal analysis, writing – original draft. **Emmanuel J. Magandi**: supervision, validation, writing – review and editing. **Sima Rugarabamu**: supervision, methodology, writing – review and editing, public health interpretation. All authors read and approved the final manuscript. Mohammed Elmogiera Fadlallh Elsayed is the guarantor.

## Funding

The authors have nothing to report.

## Disclosure

This research was conducted as part of the corresponding author's (Mohammed Elmogiera Fadlallh Elsayed) undergraduate medical degree requirements at the University of Medical Sciences and Technology.

## Ethics Statement

This study was approved by the Institutional Review Committee of Muhimbili National Hospital, Tanzania (MNH/IRB/Vol. IX/2025/031).

## Consent

Written informed consent for participation and publication of anonymized data was obtained from all participants prior to enrolment in the study.

## Conflicts of Interest

The authors declare no conflicts of interest.

## Data Availability

Data are available upon reasonable request. Deidentified participant data are available from the corresponding author (sima_luv@yahoo.com) following reasonable request and with a signed data access agreement.
